# Rational Design, Synthesis and Evaluation of γ-CD-Containing Cross-Linked Polyvinyl Alcohol Hydrogel as a Prednisone Delivery Platform

**DOI:** 10.3390/pharmaceutics10010030

**Published:** 2018-03-07

**Authors:** Adolfo Marican, Fabián Avila-Salas, Oscar Valdés, Sergio Wehinger, Jorge Villaseñor, Natalia Fuentealba, Mauricio Arenas-Salinas, Yerko Argandoña, Verónica Carrasco-Sánchez, Esteban F. Durán-Lara

**Affiliations:** 1 Instituto de Química de Recursos Naturales, Universidad de Talca, Talca 3460000, Maule, Chile; amarican@utalca.cl (A.M.); jvillase@utalca.cl (J.V.); nfuentealba@utalca.cl (N.F.); 2 Centro de Nanotecnología Aplicada, Facultad de Ciencias, Universidad Mayor, Huechuraba 8580000, Región Metropolitana, Chile; 3 Centro de Bioinformática y Simulación Molecular (CBSM), Facultad de Ingeniería, Universidad de Talca, Talca 34600000, Maule, Chile; marenas@utalca.cl (M.A.-S.); yargandona@utalca.cl (Y.A.); 4 Vicerrectoría de Investigación y Postgrado, Universidad Católica del Maule, Talca 3460000, Maule, Chile; ovaldes@ucm.cl; 5 Department of Clinical Biochemistry and Immunohematology, Faculty of Heatlh Sciences, Universidad de Talca, Talca 3460000, Maule, Chile; snunez@utalca.cl; 6 Center for Studies of Exercise, Metabolism and Cancer (CEMC), Universidad de Chile, Independencia 8380000, Región Metropolitana, Chile; 7 Departamento de Microbiología, Facultad de Ciencias de la Salud, Universidad de Talca, Talca 3460000, Maule, Chile; vecarrasco@utalca.cl; 8 Instituto de Innovación Basada en Ciencia, Universidad de Talca, Talca 3460000, Maule, Chile; 9 Biomaterials Laboratory Drug Delivery and Controlled Release, Núcleo Científico Multidisciplinario, Dirección de Investigación, Universidad de Talca, Talca 3460000, Maule, Chile

**Keywords:** drug delivery, crosslinking, prednisone, cyclodextrins, swelling, accumulative release, thermogravimetric analysis

## Abstract

This study describes the *in-silico* rational design, synthesis and evaluation of cross-linked polyvinyl alcohol hydrogels containing γ-cyclodextrin (γ-CDHSAs) as platforms for the sustained release of prednisone (PDN). Through *in-silico* studies using semi-empirical quantum mechanical calculations, the effectiveness of 20 dicarboxylic acids to generate a specific cross-linked hydrogel capable of supporting different amounts of γ-cyclodextrin (γ-CD) was evaluated. According to the interaction energies calculated with the *in-silico* studies, the hydrogel made from PVA cross-linked with succinic acids (SA) was shown to be the best candidate for containing γ-CD. Later, molecular dynamics simulation studies were performed in order to evaluate the intermolecular interactions between PDN and three cross-linked hydrogel formulations with different proportions of γ-CD (2.44%, 4.76% and 9.1%). These three cross-linked hydrogels were synthesized and characterized. The loading and the subsequent release of PDN from the hydrogels were investigated. The *in-silico* and experimental results showed that the interaction between PDN and γ-CDHSA was mainly produced with the γ-CDs linked to the hydrogels. Thus, the unique structures and properties of γ-CDHSA demonstrated an interesting multiphasic profile that could be utilized as a promising drug carrier for controlled, sustained and localized release of PDN.

## 1. Introduction

Currently, corticosteroids are the most widely used class of anti-inflammatory drugs. Their clinical effectiveness in the treatment of dermatological disorders is related to their vasoconstrictive, anti-inflammatory, immunosuppressive and anti-proliferative effects [1,2]. Among these types of drugs, we have the prednisone (PDN) (Table 1), which is a hydrophobic drug moderately soluble in water. The PDN can be delivered in cell culture systems without the need of using a carrier. When the PDN is added to a culture medium, the drug is partially dissolved in water and quickly crosses the cellular membrane, binding to the intracellular high affinity receptors [3]. Nevertheless, treatment effectiveness utilizing corticosteroids is extremely dependent on drug release at the application site because high drug concentration frequently leads to severe local and systemic adverse effects [2,4]. Therefore, given the adverse side effects caused by conventional therapies in patients, a new field—large carrier-based drug delivery systems—has emerged that fights the side effects. These types of drug delivery systems possess drug carriers such as nanoparticles, dendrimers, cyclodextrins, liposomes and hydrogels that carry the drug inside the core/pocket/scaffold [5,6]. Among these many types of drug delivery methodologies, the development of hydrogels based on natural and synthetic polymers as drug carriers has received special attention. These biomaterials present an exciting chance for designing new methods as drug delivery platforms [4,7,8].

The principal characteristic of hydrogels is its porosity, which can simply be tunable by adjusting the cross-link density in their structure and their attraction toward water molecules [10,11,12]. Moreover, the pores of hydrogels allow drugs to be loaded and then released. The benefits presented by hydrogels for drug delivery applications involve the chance for sustained release, which results in keeping a suitable local concentration of bioactive compounds over a long period [5,13,14].

Polyvinyl alcohol (PVA) hydrogels [15,16,17], dicarboxylic acids (DCAs) [18] and cyclodextrins (CDs) [19,20] are commonly used as biomaterials because they have a high biocompatibility and are FDA-approve. CDs were selected because they are able to form inclusion complexes (host-guest type) with several classes of compounds including hydrophobic drugs. The classical CDs series are constituted of six, seven and eight d-glucopyranoside units (α-CD, β-CD or γ-CD, respectively) linked by R-1,4 bonds, which forms hydrophobic cavities.

The goal of the present article was to rationally design, synthesize and characterize cross-linked polyvinyl alcohol hydrogels (cross-linked with succinic acid (SA)) to which different concentrations of γ-CD were added. These hydrogels were named γ-CDHSAs. Moreover, in this study the in vitro release capacity of γ-CDHSAs as a strategy to delivery PDN was determined. The effectiveness of γ-CDHSA to release this corticoid could be utilized as a serious alternative to treat inflammatory diseases.

## 2. Materials and Methods

### 2.1. Theoretical Section

#### 2.1.1. Building Molecular Structures

Structures of PDN, 20 dicarboxylic acids (DCAs), PVA monomer and cyclodextrins (α-CD, β-CD and γ-CD) were built using MarvinSketch program version 17.29 (For OSX), ChemAxon Ltd., Budapest, Hungary [21], considering their protonation states at pH 7.0. The geometry of their structures was optimized using the software Gaussian version 03 Inc., Wallingford, CT, USA [22] at Density Functional Theory (DFT) level with B3LYP method and 6-311G + (d,p) basis set. The DCAs (Table S1) were selected from experimental studies related with the formation of cross-linked PVA hydrogels, which used DCAs for the cross-linking process [23,24,25,26,27,28,29,30,31,32,33].

#### 2.1.2. In-Silico Calculation of Interaction Energies

A semi-empirical quantum mechanical strategy described in previous works [34,35,36] was used to calculate the interaction energy of molecule1–molecule2 complexes. Briefly, single-point energies (1SCF) for a specific molecular conformation (molecule1–molecule2 complex) and also from their isolated parts were calculated using Parameterized Method number 7 (PM7) [37] implemented in MOPAC2016 software version 16.111L (for LINUX), Colorado Springs, CO, USA [38]. The total energy (*E*) was extracted of the previous 1SCF calculation. Then, a super molecular approach was used to obtain directly the interaction energy (Δ*E*) the Equation (1):

Δ*E*_1,2_ = *E*_(molecule1–molecule2)_ − (*E*_(molecule1)_ + *E*_(molecule2)_) (1)

In the first study, molecule1 represented each one of the cyclodextrins (α-CD, β-CD and γ-CD) and molecule2 represented 100 thousand different orientations of PDN around the molecule1. Then, the average of the interaction energies calculated for each type of complex was obtained. This study allowed selecting which cyclodextrin had the best interaction by PDN, with the objective of later carrying out the experimental studies only with the selected one.

In the second study, molecule1 represented the selected cyclodextrin and molecule2 represented 100 thousand different orientations of each one of the 20 DCAs (Table S1), which were covalently attached at one end to a short PVA chain of 5 monomers long. The objective of this study was to determine the specific crosslinking agent that allows improving the affinity of the PVA hydrogel by the selected cyclodextrin. Finally, the average of the interaction energies calculated for each complex was obtained.

#### 2.1.3. Molecular Dynamic Simulation (MDS)

56 PVA chains, each 35 monomers long, were generated using the LEAP module of AmberTools17 software version 17.05 (for Linux) University of California, San Francisco, CA, USA [39]. Subsequently, using PACKMOL software version 16.070.3 (for Linux) [40] these chains were randomly distributed within a three-dimensional orthorhombic box of 90 Å × 80 Å × 100 Å (*X*, *Y* and *Z* axes). A distance of at least 5 Å was used to separate each chain from each other. The LEAP module was used to perform the crosslinking procedure according the previous work [36], which was based on a cyclic iteration scheme. Briefly, two –OH groups of two different PVA chains (separated by no more than 10 Å) were randomly selected, then, these –OH groups were covalent bonding with the –COO^−^ groups of SA, the geometry of the modified polymer system was formatted and optimized (in order to avoid steric hindrance) using the Antechamber software package version 1.27 (for Linux) [41] and the steepest descent algorithm and the Universal Force Field (UFF) implemented in Openbabel software version 2.3.1 (for Linux) [42]. Finally, considering that the polymer matrix had a total of 1960 PVA monomers, the cyclic iteration scheme was repeated until to incorporate 392 SA inside the PVA polymer matrix, in order to model the 10:2 proportion of PVA:SA.

The previous PVA polymer matrix was used to generate four models of hydrogel formulations with different proportions of γ-CD linked to their structures (approximately, 0%, 2.5%, 5% and 10%). 0, 6, 12 and 24 γ-CD molecules were linked in each hydrogel using LEAP module of AmberTools17 software version 17.05 (for LINUX) University of California, San Francisco, CA, USA [39]. These systems were named: γ-CDHSA0, γ-CDHSA1, γ-CDHSA2 and γ-CDHSA3.

30 PDN molecules were randomly added around each cross-linked PVA matrices (considering a separation distance of 10 Å) using PACKMOL software version 16.070 (for Linux) [40]. The final systems were added in the center of solvent boxes of the following size: 160 Å, 160 Å, 160 Å (axes *X*, *Y*, *Z*, respectively) using the System Builder Module of Desmond/Maestro software academic release 2017-4 (for Linux) [43] subsequently the boxes were solvated with TIP3 water molecules. Sodium and chloride ions were added to the aqueous phase to mimic physiological conditions (0.15 M NaCl). The default relaxation protocol implemented in Desmond/Maestro software academic release 2017-4 (for Linux) DE Shaw Research, NY, USA [43] was used. Briefly, this protocol involves a series of steps: first the molecular systems were energy minimized using a steepest descent algorithm switching on and switching off restraints over heavy atoms, then, a series of four short NVT (constant Number, Volume and Temperature) and NPT (constant Number, Pressure and Temperature) simulations (of 12, 12, 12 and 24 ps, respectively) were performed retaining restraints to finally perform an unrestrained simulation. The final simulations involving the following parameters: a NPT ensemble with a relaxation time of 1.0 ps was used, in which the temperature was kept at 300 K using the Nosé–Hoover chain method. The Martyna–Tobias–Klein (MTK) barostat method was used to keep the pressure fixed at 1.0 bar (considering an isotropic coupling and a relaxation time of 2.0 ps). The equations of motions were incorporated using the RESPA integrator considering a time step of 2.0 fs for bonded and near interactions and a time step of 6.0 fs for far interactions. A cutoff radius of 9.0 Å was employed for non-bonding interactions such as electrostatic and Van der Waals interactions. The parameter and charges of the systems were automatically assigned using the OPLS_2005 force field. Finally, the four MDS were run for about 100 ns each.

In order to perform the intermolecular analysis, 1000 frames were extracted from MDS results. For each hydrogel in the MDS, the Solvent Accessible Surface Area (SASA) [44], the Radius of Gyration (RGYR) [45] and the number of waters inside the hydrogel were calculated. The above allowed observing the displacement, behavior and structural integrity of the hydrogel. The capture of PDN by γ-CDHSA was calculated in order to analyze the hydrogel-drug interaction and the procedure was executed using TCL scripts implemented in VMD software version 1.9.2 (for Linux) [46], a distance of 4.5 Å between PDN and γ-CDHSA atoms was considered. Gnuplot software version 5.0 [47] was used to plot the results of SASA, RGYR and capture calculations. Finally, the intermolecular interactions that occur between γ-CDHSAs and PDN were analyzed using the BIOVIA-Discovery Studio Visualizer (BIOVIA-DS Visualizer) version 4.5. (for Linux) Accelrys Software Inc., San Diego, CA, USA [48].

### 2.2. Experimental Section

#### 2.2.1. Materials

Polyvinyl alcohol (PVA) 30–60 KDa, succinic acid (SA), γ-cyclodextrin polymer (γ-CD), NaHCO_3_, (3,4,5-dimethylthiazol2-yl)-2–5-diphenyltetrazolium bromide (MTT), acetonitrile (HPLC grade) and prednisone (PDN) analytical standards were purchased from Sigma-Aldrich (St. Louis, MO, USA), HCl and methanol (HPLC grade) were purchased from Merck (Darmstadt, Germany). All solutions were prepared using MilliQ water.

#### 2.2.2. Synthesis of γ-CDHSAs

**Figure 1:**
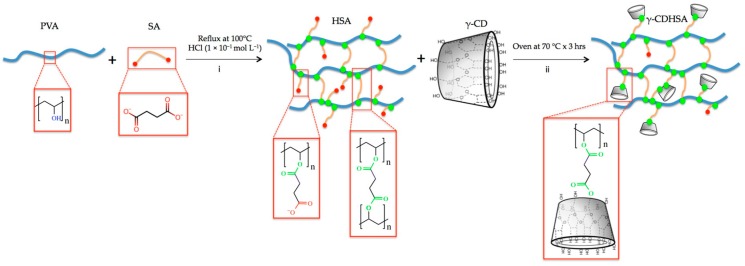
Schematic representation of γ-CDHSA (Polyvinyl alcohol hydrogel cross-linked with succinic acid and containing γ-cyclodextrin) synthesis, which were divided into two steps. First step (pre-hydrogel): polymerization by esterification of PVA (Polyvinyl alcohol ) with SA (Succinic acid) in the presence of HCl as a catalyst. Second step: polymerization by esterification of PVA-SA hydrogel with γ-CD (γ-cyclodextrin).

Three γ-CDHSAs with different percentages *w*/*w* % of γ-CD were synthetized. The syntheses of these formulations were carried out through the esterification of PVA with SA according to the modified method from Schanuel et al. [6] (Figure 1). Briefly, the reactions were performed by mixing aqueous solution of PVA with an aqueous solution of SA (20 wt %) in presence of 1 × 10^−1^ mol L^−1^ HCl (pH 1). The reaction was performed under reflux at 100 °C in a necked flask with magnetic agitation. After 3 h, the reaction mixture was divided into three equal parts and to each one a different amount of γ-CD was added. The final concentration of γ-CD for each formulation was 2.44, 4.76 and 9.10 wt %, designated as γ-CDHSA1, γ-CDHSA2 and γ-CDHSA3, respectively. After that, each reaction was put in an oven at 70 °C for another 3 h to complete the crosslinking. Then, the γ-CDHSA1, γ-CDHSA2 and γ-CDHSA3 were washed three times with NaHCO_3_ for removing the excess acid. Finally, the hydrogels were lyophilized in order to obtain the xerogel.

#### 2.2.3. Swelling Studies

Dried hydrogel membrane discs (0.4–0.5 mm thickness, 1 cm diameter) were left to swell in phosphate buffer saline (PBS) (pH 7.4), acetate buffer (pH 4.0) and ammonium chloride buffer (pH 10.0) at 25 °C. Swollen gel was removed from the swelling medium at regular time intervals, between 0 and 47 h and superficially dried with filter paper. Then, it was weighed and placed in the same bath. The measurements were continued until a constant weight was reached (Equation (2)):

(2) $$\%W=\frac{M_{h}-M_{x}}{M_{h}}\;\times \;100$$

where, %W is swelling index, *M_h_* and *M_x_* are the mass of the swollen hydrogel and the xerogel, respectively. To analyze the rate of water absorption, the water-intake process was evaluated by the determination of the swelling ratio of the hydrogel at desired time intervals as previously described. With the results, a screening analysis was carried out to determine the statistical significance of the experimental variables, coding them between −1 and 1, so that they had the same statistical weight.

#### 2.2.4. Infrared Spectroscopy

Fourier-Transform Infrared (FT-IR) spectra of γ-CDHSA1, γ-CDHSA2 and γ-CDHSA3 were recorded on a Nicolet Nexus 470 spectrometer (Thermo Scientific, Waltham, MA, USA) within the 4000–400 cm^−1^ spectral intervals. All spectra were obtained in KBr pellets from an average of 32 scans with 4 cm^−1^ resolution.

#### 2.2.5. Thermogravimetric Analysis (TGA)

TGA was carried out using a TGA analyzer Q500 (TA Instruments, New Castle, DA, USA). The sample was ramped from 30 to 600 °C at 10 °C min^−1^ with gas flow rate at 60 mL min^−1^ in synthetic air and nitrogen atmosphere, respectively. The mass remaining was recorded throughout the experiment.

#### 2.2.6. Scanning Electron Microscopy (SEM) Analysis

The samples were cut and loaded in the copper stub. Then, they were stained with 0.7% (*w*/*v*) phosphotungstic acid, washed and air-dried. The samples were examined in SEM mode, in a Low-Voltage Electron Microscope (at a nominal operating voltage of 5 kV) LVEM5 (Delong Instruments, s.r.o., Brno, Czech Republic).

#### 2.2.7. Loading of Prednisone γ-CDHSA1, γ-CDHSA2 and γ-CDHSA3 in Model Solutions

The loading study of PDN by γ-CDHSA1, γ-CDHSA2 and γ-CDHSA3 was evaluated determining the difference in mass using the following Equation (3):

Drug content (mg) = X_0_ − X_PDN_ (3)

where X_0_ is the mass of xerogel without PDN and X_PDN_ is xerogel with PDN. A model solution of 0.22 mg mL^−1^ of PDN in buffer phosphate saline (PBS), pH 7.4 was used for all assays of loading by γ-CDHSAs. 50 mg of each γ-CDHSAs was used per test, using 10 mL of model solution for each experiment. According to equilibrium swelling ratio (ESR) analysis, the equilibrium was reached at 5 h and the γ-CDHSAs were incubated in a thermostat-containing reciprocal shaker (120 rpm) at 22 °C for 6 h.

#### 2.2.8. Drug Release Studies of γ-CDHSAs

The drug-loaded supramolecular hydrogels γ-CDHSA1, γ-CDHSA2 and γ-CDHSA3 were prepared as shown in Table 2. Briefly, these formulations were loaded with a unique concentration of PDN solution dissolved in the PBS (0.22 mg mL^−1^). The hydrogel formulation without drugs was prepared as blank. Then, 50 mg of the membrane of each formulation were loaded into a 10 mL tube and 5 mL of PBS (pH 7.4) were poured over the formulation as a release medium. The tubes were transferred to a shaker incubator water bath (Farazteb, Iran) at 22 °C ± 0.1 °C and shaken at 40 ± 2 rpm. At sampling times 0, 0.5, 1, 2, 3, 4, 6, 8, 10, 13 and 25 h, a 1 mL aliquot of release medium was withdrawn to determine the amount of drug released and the aliquot was replaced by fresh PBS. The PDN samples were analyzed by a Perkin Elmer series 200 HPLC system (Norwalk, CT, USA) with a UV-Vis detector and a C-18 Kromasil 100-5-C18 (250 mm × 4.6 mm i.d. × 5 μm) column was used for the analysis of eluents. 20 μL of sample was injected into the HPLC apparatus. An isocratic elution with 20 mM PBS/Acetonitrile (65:35, *v*/*v*) at a flow rate of 1.0 mL min^−1^ was used as the mobile phase. The eluents were monitored at 254 nm by absorbance detection at room temperature.

PDN release from the hydrogels was determined by applying the amounts of released and absorbed PDN to the following relationship (Equation (4)):

(4) $$\mathrm{PDN}\;\mathrm{cumulative}\;\mathrm{release}\;\left(\%\right)\;=\;\mathrm{Amount}\;\mathrm{of}\;\mathrm{released}\;\mathrm{PDN}\;\times \frac{100}{\mathrm{Amount}\;\mathrm{of}\;\mathrm{absorbed}\;\mathrm{PDN}}$$

#### 2.2.9. Cytotoxicity and Cell Viability

Viability of fibroblasts was assessed using MTT assay according to the protocol of Mossman et al. [8,20]. Briefly, the cells were seeded in 24-well plates (1.6 × 10^4^ cells per well). Then, 5 μL of cells and 150 μL of Dulbecco’s Modified Eagle Medium (DMEM)-High medium were added and incubated for 24 h at 37 °C in 5% CO_2_. Then, the medium was replaced by 100 μL of fresh DMEM-High per well containing different amounts of γ-CDHSA1, γ-CDHSA2 and γ-CDHSA3, specifically, 500 μg mL^−1^, 1000 μg mL^−1^, 1500 μg mL^−1^, 2000 μg mL^−1^ and 2500 μg mL^−1^ per formulation. Fresh medium without hydrogel was used as a control. Cell viability was evaluated after 24 h by the MTT assays. Specifically, 5 μL of MTT solution (3 mg mL^−1^ in PBS) and 50 µL of fresh medium were added to each specimen and incubated for 4 h in the dark at 37 °C; formazan crystals were then dissolved in 100 µL dimethyl sulfoxide (DMSO) and incubated for 18 h. Supernatant optical density (o.d.) was evaluated at 570 nm (Spectrophotometer, Packard Bell, Meriden, CT, USA). Untreated cells were taken as a control with 100% viability. The cell cytotoxicity of supramolecular hydrogel was defined as the relative viability (%), which correlates with the number of liable cells compared with negative cell control (100%).

#### 2.2.10. Statistical Analysis

A customized experimental design (based on 2^N^ design) was performed to determine the best experimental conditions for release of PDN by γ-CDHSAs. The values were coded between −1 and 1 to give the same statistical weight to the experimental variables. Subsequently, the PDN was released in the optimal conditions predicted by the model. These results were expressed as the means ± Standard Deviation (S.D.) from three replicates in order to minimize the experimental error.

## 3. Results

### 3.1. In-Silico Interaction Energy Study

The cyclodextrins α-CD, β-CD and γ-CD were evaluated as possible candidates to improve the affinity of PDN for PVA hydrogels. The average of interaction energy of each CD/PDN complex was calculated using semi-empirical quantum mechanical calculations. The obtained values were −3.76 ± 0.08, −3.70 ± 0.05 and −2.24 ± 0.04 kcal mol^−1^ for γ-CD/PDN, β-CD/PDN and α-CD/PDN complexes, respectively.

**Figure 2:**
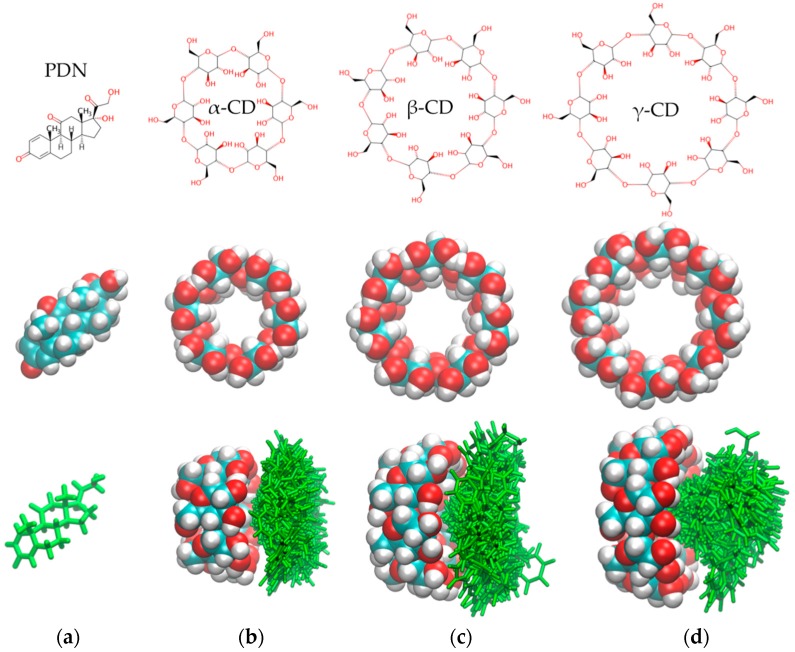
(**a**–**d**) the top and middle images show the molecular structures of PDN (Prednisone), α-CD, β-CD and γ-CD; (**b**–**d**) the bottom images show the spatial distribution of the 100 conformations of the best interaction energy for the complexes: α-CD/PDN, β-CD/PDN and γ-CD/PDN.

Figure 2b–d (the bottom images) depicts side views of the spatial distributions for the 100 conformations of the lowest interaction energies obtained with the semi-empirical calculations, where it is possible to appreciate that γ-CD (Figure 2d, the bottom image) was able to form PDN inclusion complexes better than β-CD (Figure 2c, the bottom image) and α-CD (Figure 2b, the bottom image)—in the latter, PDN failed to penetrate the α-CD cavity, mainly due to the steric hindrance of the smallest cavity. Therefore, γ-CD could play a key role in improving the retention capacity of the PVA hydrogels by the PDN molecules.

**Figure 3:**
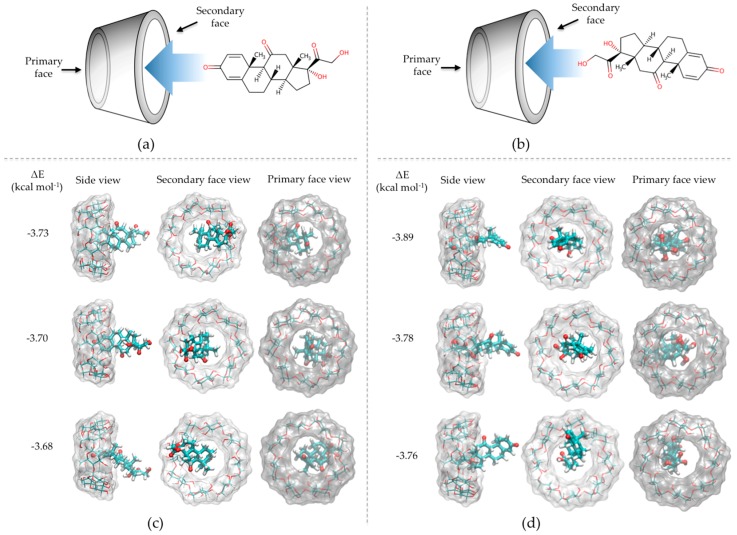
Representative scheme for the approaches that led to the formation of the γ-CD/PDN complex: (**a**) the inclusion processes A; (**b**) the inclusion process B; (**c**,**d**) Side, secondary and primary face views of the 1:1 inclusion complexes with the lowest interaction energy.

When the PDN geometries inside the γ-CD cavities were compared, it was observed that the γ-CD/PDN complexes were generated in two approaches, inclusion process A and B (Figure 3). The inclusion process A was performed through the =O groups (of 2,5-cyclohexandienone) of PDN pointing toward into the secondary face of γ-CD hydrophobic cavity (Figure 3a) and the inclusion process B, where the –OH groups from the other end of PDN were those who entered the interior of the cavity (Figure 3b). This latter obtained 80% of the inclusion cases, therefore, this indicates that the precise size of the γ-CD cavity provides the most favorable spatial geometry to generate the γ-CD/PDN complex. Figure 3c,d shows the views (side, secondary and primary face) of the three γ-CD/PDN complexes with the lowest interaction energy for A and B inclusion processes, respectively.

Once γ-CD was selected, the evaluation process of a series of DCAs was performed, in order to obtain the best candidate that would allow to implement efficiently the PVA crosslinking procedure and at the same time generate a stable polymer matrix that allows the addition of γ-CD in its structure. Table 3 shows the values of the average interaction energies calculated between γ-CD and each DCA covalently bonded to a short PVA (PVAc). These calculations allowed for quickly evaluating the contribution of the structural features of each DCA (Table S1) in the obtained interaction energy values. The results showed that succinic acid (SA), terephthalic acid (TA) and aspartic acid (AA) had the best average interaction energies (−2.67 ± 0.03, −2.70 ± 0.05 and −2.83 ± 0.08 kcal mol^−1^, respectively) and therefore, they would be good candidates for the process of crosslinking the PVA hydrogel. Finally, SA was the candidate chosen due to its good hydrophobicity and easy manner to produce a PVA hydrogel.

### 3.2. Molecular Dynamics Simulations (MDS) Studies

MDS studies were performed in order to understand the molecular behavior and the interactions between PDN molecules and four cross-linked PVA hydrogels to whom different proportions of γ-CD were added to their structures 0%, 2.5%, 5% and 10% (γ-CDHSA0, γ-CDHSA1, γ-CDHSA2 and γ-CDHSA3, respectively). The hydrogels and PDN molecules were immersed in water boxes mixed with sodium and chloride ions (0.15 M NaCl) in order to mimic physiological conditions. Finally, four MDS were carried out for about 100 ns of simulation.

**Figure 4:**
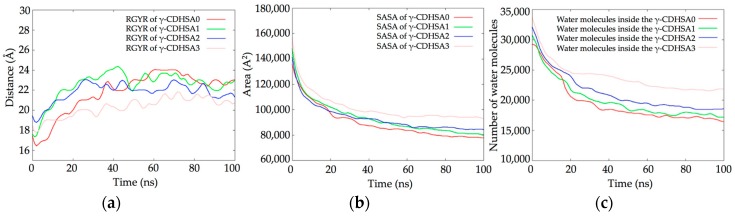
(**a**) Radius of Gyration (RGYR) and (**b**) Solvent Accessible Surface Area (SASA) plots of the four hydrogels. (**c**) Number of water molecules inside the hydrogels.

Figure 4a shows calculated RGYRs. It is possible to observe that as the concentration of γ-CD in the structure of the hydrogels increases, they showed a slight reduction in their RGYR. The difference between γ-CDHSA0 and γ-CDHSA3 was just 2 Å, (20 and 22 Å, respectively). The SASA values allowed to quantify the accessible area of each hydrogel that interact with both the solvent and the PDN molecules (Figure 4b). γ-CDHSA0 showed an approximate SASA of 78,000 A^2^, which was incremented in 4%, 8% and 20% as the concentration of γ-CD in their structures increased, 81,000 A^2^, 84,000 A^2^ and 93,000 A^2^ (γ-CDHSA1, γ-CDHSA2, γ-CDHSA3, respectively).

The role of γ-CD in the hydration in the conformational structure of each hydrogel was determined by counting the number of water molecules located no more than 3.0 Å from the hydrogel surface plus all the rest of the water molecules located inside each hydrogel. Figure 4c shows the behavior of the water molecules inside the hydrogels during the MDS trajectories. It is possible to observe that the incorporation of CD in the hydrogel structure allowed him to interact with at least 6%, 15% and 35% more water molecules.

**Figure 5:**
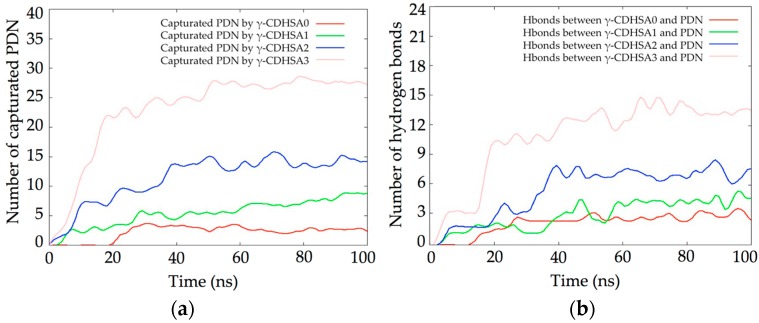
(**a**) Plot of PDN molecules captured by the four hydrogels; (**b**) number of hydrogen bonds identified during the simulation.

The capture of PDN by each hydrogel during the 100 ns of simulation is shown in Figure 5a. It was considered a contact distance of 4.5 Å between the γ-CDHSAs backbone and PDN molecules. γ-CDHSA1 only stably captured 2 and 3 molecules of PDN, this due to the hydrophobicity of the drug and therefore less affinity for the hydrogel. As the γ-CD was added, the affinity of the drug for the hydrogel increased proportionally. γ-CDHSA3, which had a maximum of 24 γ-CDs in its structure, managed to capture between 26 and 27 PDN molecules. This would indicate that drugs interacted with both γ-CD and the PVA skeleton of the hydrogel.

When hydrogen bonds between each hydrogel and the PDN molecules were analyzed (Figure 5b) it was observed that there was not a direct relationship between the capture of PDN and the number of hydrogen bonds. In the light of the foregoing, this would indicate that due to the hydrophobic nature of PDN, it would tend to interact mainly through van der Waals interactions in the hydrophobic regions of the hydrogels.

**Figure 6:**
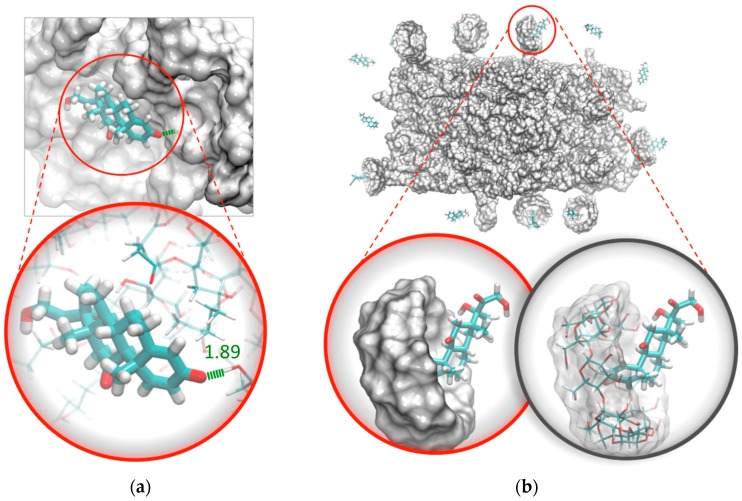
PDN interaction with: (**a**) surface pocket of PVA hydrogel through hbonds and (**b**) with the hydrophobic cavities of γ-CD added to the hydrogel.

MDS studies allowed observing the binding interactions between PVA hydrogels and PDN molecules. Figure 6 shows how the =O group of the 2,5-cyclohexandienone of PDN (Figure 6a) was able to interact through hbonds formed between –OH from PVA chains located on the surface of the PVA hydrogel. In Figure 6b, it’s possible to observe a greater proportion of PDN interacting with the hydrophobic cavities of γ-CD.

### 3.3. Preparation of γ-CDHSAs

The synthesis of γ-CDHSAs was performed as is represented in Figure 1 (methodology Section 2.2.2). Briefly, the hydrogels were prepared using polymerization by esterification in the presence of HCl as a catalyst. Once the pre-hydrogel was formed, it was added the γ-CD, where the hydroxyl groups of γ-CD were esterified with carboxyl groups still available from SA. The characterization analysis from FT-IR and TGA confirmed the conjugation between γ-CD and SA into the hydrogel. Considering the previously reported [6], a crosslinking degree of 10:2 of PVA:SA was used, which was kept constant due to its better efficiency since it presented a good porosity, allowing a greater stability [6]. In contrast, the γ-CD content was modified as depicted in Table 2.

### 3.4. ESR

**Figure 7:**
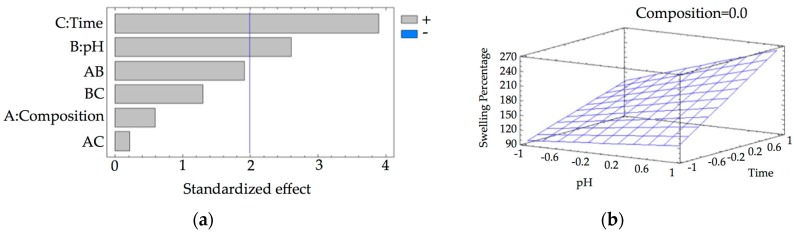
(**a**) Standardized Pareto chart for Hydrogel Swelling percentage (Where: A, CD composition; B, pH; C, time; AB, AC and BC, interaction. The line represents the critical *t*-value, 95% confidence). (**b**) Estimated response surface.

Table S2 shows the results of the equilibrium-swelling ratio in percentage of the hydrogel under the experimental conditions described above. Figure 7 shows the Pareto chart and the estimated response surface.

From the Pareto chart (Figure 7a) it is determined that all the variables in the study exercised a positive influence. The statistically significant variables corresponded to pH and time and the interaction between γ-CD composition and pH. On the surface of estimated response (Figure 7b), it is observed that as the pH increases, the swelling percentage also increases. In the same way, as the contact time increases, the swelling percentage also increases, although in a much more marked way. Considering the statistical significance of the variables, the regression equation of the model is (Equation (5)):

Swelling Percentage = 162.11 + 30.45 · B + 51.85 · C + 19.20 · A · B (R^2^ = 18.05) (5)

**Figure 8:**
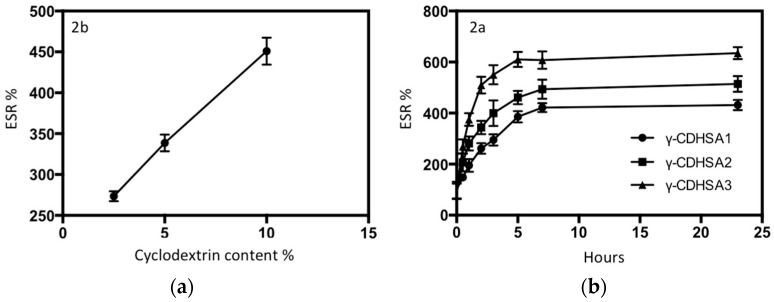
(**a**) Dependence of the ESR of γ-CDHSAs hydrogels on the amount of γ-CD; (**b**) ESR of γ-CDHSA1, γ-CDHSA2 and γ-CDHSA3 at pH 7.4, with respect to time.

Although the γ-CD proportion is not a statistically significant variable compared to the other experimental variables, it has a positive influence on the swelling percentage. The incorporation of γ-CD into the hydrogels led to an increase in ESR as depicted in Figure 8a, which could be justified by the multiple hydroxyl groups on γ-CD forming hydrogen bonds with water molecules [49].

Considering that it is desired to evaluate the release of PDN under physiological pH conditions, the following experiments were carried out: the assays were carried out with the aim of evaluating the swelling capacity of the prepared hydrogels using different γ-CD proportions at pH 7.4 at room temperature. The swelling index for the three different formulations is shown in Figure 8b. This figure shows that the swelling index increased significantly with time for the set of γ-CDHSAs. For all γ-CDHSAs, the swelling index in the first part increased rapidly and afterwards slowly. This behavior was due to the hydrogels reaching maximum constant swelling. The γ-CDHSA1, γ-CDHSA2 and γ-CDHSA3 reached the swelling equilibrium (zero order) at about 5 h. γ-CDHSA1 showed a swelling behavior with a value of 300% approximately.

**Figure 9:**
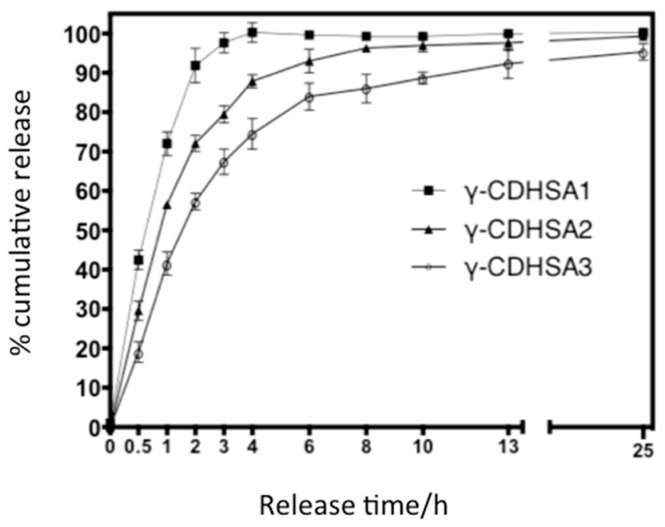
PDN release profile from PDN-absorbed γ-CDHSAs hydrogels in PBS solution, pH 7.4.

By contrast, γ-CDHSA2 and γ-CDHSA3 were higher, 450% and 600%, respectively, as depicted in Figure 9. As has been described in other works [50,51], the swelling index depends on the nature of the polymer, the average molecular weight, the degree of crosslinking, the rigidity of the polymer chain and the network mesh size as well as external conditions, such as pH and temperature [50,51], Moreover, the swelling index observed could be related with the absorption mechanism, which is determined by the diffusion process of water into the pores of hydrogel [52].

### 3.5. Drug Loading and In Vitro Release Behavior of γ-CDHSAs

Table 4 shows the amount of PDN loaded into the γ-CDHSAs composite hydrogels. It was observed that there is no statistically significant difference between the results obtained.

**Figure 10:**
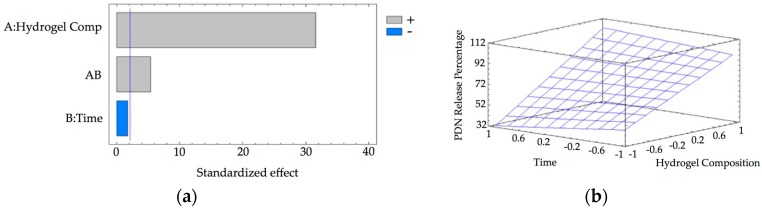
(**a**) Standardized Pareto chart for PDN release percentage due to hydrogel treatment (Where: A, Hydrogel composition; B, time of release; AB, interaction. The line represents the critical *t*-value, 95% confidence); (**b**) Estimated response surface.

Table 5 shows the results of PDN release experiments. Based on these results, the release behavior of PDN from γ-CDHSAs in PBS is presented in Figure 10. The Pareto chart (Figure 10a) shows that γ-CDHSAs’ composition and their interaction with time are statistically significant, exerting a positive influence on the PDN release percentage. Time of release was not statistically significant. The estimated response surface (Figure 10b) shows that the percentage of release increased when the composition of γ-CD in hydrogel rises, reaching a maximum value at the end of the interval.

Considering the statistical significance of the studied variables, the regression equation of the model is (Equation (6)):

*PDNReleasePercentage* = 69.95 + 30.66 · A + 5.53 · A · B (R^2^ = 64.77) (6)

The optimum experimental conditions for the release of this compound were as follows: Percentage of γ-CD in hydrogel composition of 9.1%. Although time of release is not a statistically significant variable, it is recommended to use the longer contact time (48 h) to ensure a better release of PDN.

Among steroidal drugs, PDN is well known for its capability to undergo complexation with γ-CD [6,19]. The dried γ-CDHSAs’ membranes were immersed in an aqueous solution (PBS) of PDN (0.22 mg mL^−1^) at 25 °C for drug loading. It was found that the hydrogels could be loaded with PDN to equilibrium after 6 h of immersion according to ESR (Figure 8b). Table 5 depicted the equilibrium amount of PDN loaded in the γ-CDHSAs, the γ-CD content in the hydrogels affected the amount of PDN loaded in γ-CDHSAs, where γ-CDHSA1, γ-CDHSA2 and γ-CDHSA3 hydrogel achieved an absorption 8.36, 9.02 and 10.1 mg per gram of dried hydrogel, respectively. Therefore, the amount of PDN absorbed by the γ-CDHSAs at the saturated time depended on the γ-CD content of the hydrogels. This could be attributed to the formation of inclusion complexes between the hydrophobic γ-CD groups and PDN molecules [15,20]. Consequently, the formation of inclusion complexes could increase the affinity of the γ-CDHSAs hydrogel network for PDN molecules. Therefore, this study has confirmed the encapsulation abilities of the γ-CDHSAs by PDN.

In vitro PDN cumulative release from the PDN-absorbed γ-CDHSAs was explored by monitoring the amounts of cumulative released PDN from a hydrogel-PBS mixture at 25 °C as a function of time. The PDN cumulative release (%) profiles are depicted in Figure 9; the three γ-CDHSAs offered a burst release into the medium up to a release time of 2 h, at which the 91.8%, 72.1% and 56.2% of PDN had been released from γ-CDHSA1, γ-CDHSA2 and γ-CDHSA3 hydrogels, respectively. According to this result in the first stages of release, it is produced phenomenon termed “burst effect” [53] of PDN release was produced. This abrupt release could be attributed to the remaining free PDN on the hydrogel surface and interior and/or PDN weakly interacting with γ-CDHSAs hydrogels through hydrogen bonding (Figure 6a). After this initial fast release profile, γ-CDHSAs showed a slower and steadier PDN release into the medium for all cases (zero-order). On the other hand, when it increased the γ-CD content in the hydrogel, the release speed decreased as depicted in Figure 9. Perhaps, this is due to diverse factors that could be involved in the complex formation of γ-CDHSAs with PDN: hydrophobic-hydrophobic (van der Waals) interactions between the hydrophobic moiety of the PDN molecules and hydrophobic γ-CD cavity in γ-CDHSAs (Figure 6b), hydrogen bonding between the polar functional groups contained in both PDN and γ-CDHSAs and hydrogen bonding between the polar moieties of PDN and high-energy water molecules released from the hydrogel γ-CD cavity during complex formation [20,54]. Therefore, with the unique and tunable characteristics of γ-CDHSAs in relation with γ-CD content and the nature of the designed hydrogel, the exclusive drug release profiles observed for the present formulations indicate their efficacy in controlled drug delivery applications.

### 3.6. FTIR Analysis

The width and intensity of spectral bands, as well as the peak positions, are very sensitive to environmental changes and the conformations of the hydrogel. It is well known that this technique under the work condition described in the experimental section is it not quantitative, for that reason we only discussed one formulation, specifically the γ-CDHSA2. In addition, the FTIR spectra of PVA and γ-CD have already been described in the Spectral Database for Organic Compounds SDBS [55], for that reason, we did not report it in this manuscript.

**Figure 11:**
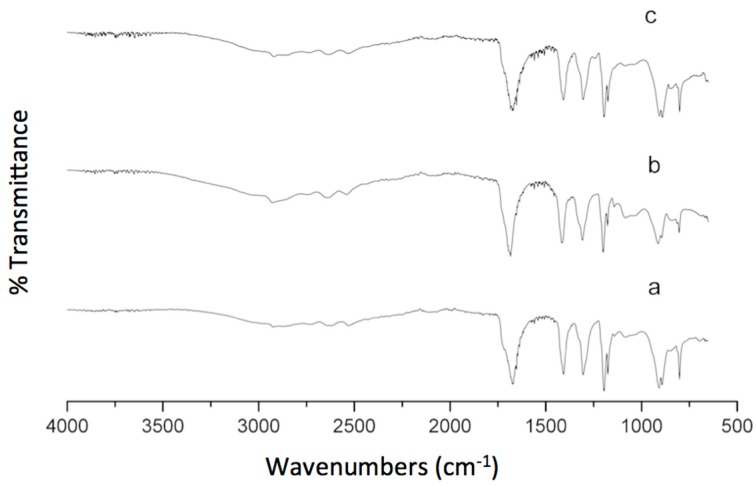
FTIR spectra of (a) γ-CDHSA1, (b) γ-CDHSA2 and (c) γ-CDHSA3.

The spectra of the PVA and γ-CD exhibited the typical absorption peaks around 3350, 2930, 1158 and 1090 cm^−1^ that are attributed to the stretching vibrations of –OH, –CH_2_, coupled –COC and coupled CO/CC, respectively. The IR spectrum of γ-CDHSA2 (Figure 11) demonstrates all characteristic absorption bands of the components that conform the hydrogel. However, the disappearance of the signals around 1096 and 920 cm^−1^, which correspond to coupled –C–O stretching and –O–H in-plane bending vibrations reported for the PVA and the appearance of the new peak at 1200 cm^−1^ is attributed to the –C–O–C stretching of the ester linkage formed due to crosslinking, confirming the formation of a SA cross-linked PVA polymer. Thus, the large bands observed centered around 3000 cm^−1^ are linked to the stretching –O–H from the intermolecular and intramolecular hydrogen bonds, the lower wavenumbers of this band are another indicator for the formation of the hydrogel. In addition, the vibrational bands observed between 2840 and 3000 cm^−1^ refer to the stretching –C–H hydrogel backbone. Moreover, at 1669 cm^−1^ we found the widest and most intense signal presented in the γ-CDHSA2 spectra, which was assigned to the asymmetrical stretching vibration of –COO groups, suggesting that new hydrogen bonds are formed between the –COOH groups of SA and the –OH groups present in the PVA and γ-CD.

It is important to note that the signal corresponding to the carboxylic group from SA was overlapped due to the great intensity of this band. The above results obtained by FTIR indicate the cross-linking of PVA was due to the ester linkage formed between SA and PVA in the presence of γ-CD.

**Figure 12:**
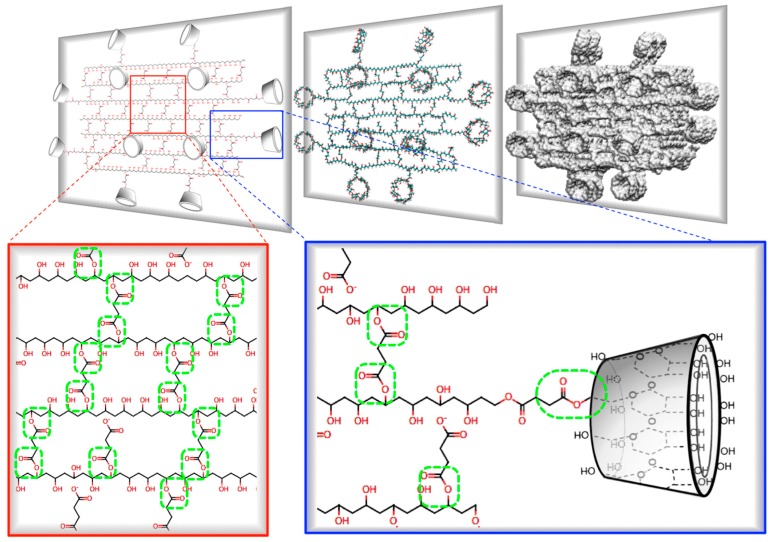
Schematic representation of a cross-section of a γ-CDHSA hydrogel, where it is possible to appreciate the ester bonds formed between PVA chains and SA (crosslinking molecule), as well as between –COO^−^ groups of SA and the –OH groups mainly located on the primary face of γ-CD.

Figure 12 shows the schematic representation of a cross-section of a γ-CDHSA hydrogel, where it is possible to appreciate the covalent ester bonds (Figure 12, green circles) formed between PVA chains and SA (crosslinking molecule), as well as between SA (partially bound at one end with PVA) and the –OH groups mainly located on the primary face of γ-CD.

### 3.7. Thermogravimetric Analysis

**Figure 13:**
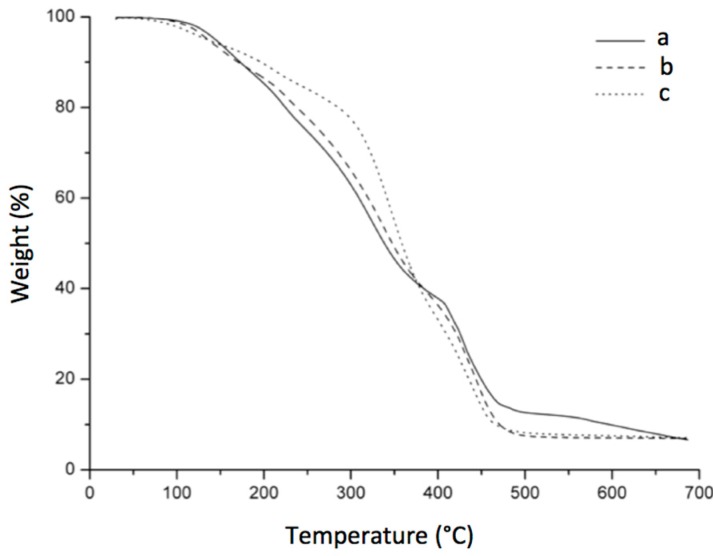
TGA (Thermogravimetric ) thermograms of (**a**) γ-CDHSA1, (**b**) γ-CDHSA2 and (**c**) γ-CDHSA3.

To verify influence of the structure and composition on thermal degradation of the obtained hydrogels, the thermal stability was determined by thermogravimetric analysis in a temperature range of 30 °C up to 700 °C. The weight loss (TG) curves for the different feed compositions of prepared hydrogels are presents in Figure 13. TGA analysis revealed the thermal effects of PVA hydrogels with the presence of γ-CD at different concentrations. All the samples exhibited four main weight loss steps. We can see that the main decomposition of the hydrogel starts at around 322, 336 and 343 °C for γ-CDHSA1, γ-CDHSA2 and γ-CDHSA3, respectively. It is important to note that these temperatures are intermediate between the decomposition temperatures of PVA (393 °C) and γ-CD (346 °C) [56,57]. This result suggests that the PVA polymer chains are included inside the γ-CD channels and can improve the thermal stability of the host CD. The obtained data agree with the results reported by Hernández et al. [58]. Another three weight loss were observed in the TGA curves. The first weight loss observed at around 120 °C with a loss mass from 5 to 12%, corresponds to weakly physisorbed water. The second stage in the range 200–300 °C was attributed to the degradation of SA [59]. Finally, the 30% weight loss in the range 400 to 600 °C is due to the decomposition of the PVA side chains.

### 3.8. SEM Analysis: Sample Preparation and Viewing

**Figure 14:**
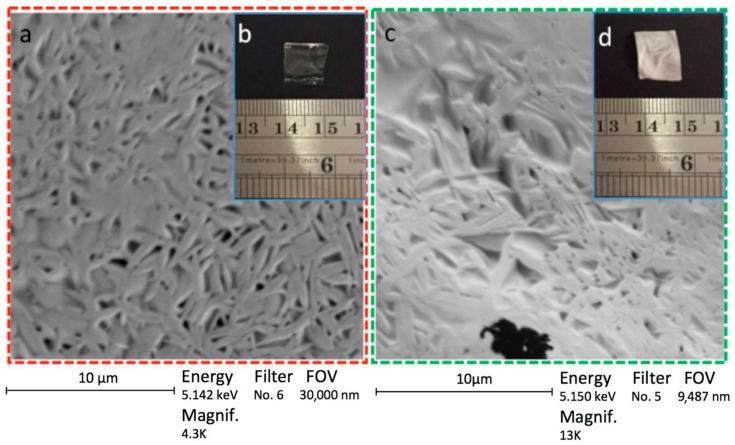
SEM micrographs (**a**) and photographs (**b**) of a small fragment of xerogel γ-CDHSA1 without PDN. SEM micrographs (**c**) and photographs (**d**) of a small fragment of xerogel γ-CDHSA1 with PDN. All hydrogels were lyophilized after the swelling process.

SEM and photographs were used to determine the morphology of the dried samples. These methods are enabled to obtain differences in morphology in both stages of hydrogel; γ-CDHSA1 without PDN and γ-CDHSA1 with PDN are depicted in Figure 14. This same figure shows SEM micrographs (Figure 14a) and photographs (Figure 14b) of the dried samples of the γ-CDHSA1 without PDN. Moreover, Figure 14 shows SEM micrographs (c) and photographs (d) of the dried samples of the γ-CDHSA1 with PDN. On the SEM micrograph of the γ-CDHSA1 without PDN, the smooth walls and an assembly of marked fiber networks can be appreciated. Moreover, it is possible to observe the high 3D porous structure with well-defined shapes, exhibiting some spread in pore size. The supramolecular hydrogel displayed a porous structure that could be attributed to the crosslinked process. This kind of structure with good permeability could play a key role to promote the PDN diffusion throughout the pores to finally form an inclusion complex with γ-CDs inside of the supramolecular hydrogel.

Figure 14b,c shows the photograph of γ-CDHSA1 without and with PDN, respectively. The presentation of γ-CDHSA1 without PDN was transparent unlike the whitish appearance of γ-CDHSA1 with trapped PDN.

### 3.9. Evaluation of γ-CDHSAs Cytotoxicity

**Figure 15:**
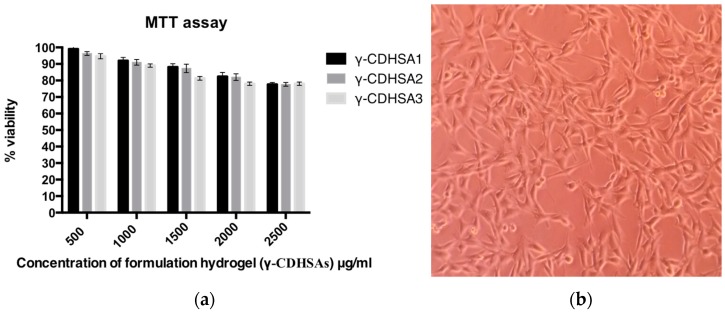
(**a**) Percentage of cell viability, obtained from the MTT assay of the L929 fibroblast cells with respect to a negative control (without supramolecular hydrogel); (**b**) Fibroblasts photograph co-cultured with 2500 μg mL^−1^ of supramolecular hydrogel γ-CDHSA3 (magnification 100×).

MTT assay was performed to quantitatively characterize fibroblast cell survival in this study. The cytotoxicity of the sterilized supramolecular hydrogels (γ-CDHSA1, γ-CDHSA2 and γ-CDHSA3) was analyzed by cell viability assay using L929 fibroblasts cells after 24 h. Figure 15a exhibited fibroblasts’ cell viability, co-cultured with different concentrations of supramolecular hydrogels (between 500–2500 μg mL^−1^). As shown in Figure 15a, with an abrupt increase of hydrogel amount, fibroblasts cell viability decreased slightly, the viability ranged between 99% and 77%. This assay provided evidence that the hydrogels were able to maintain cell viability over 77% despite being exposed to high concentrations of hydrogels. Figure 15b shows a microphotograph of fibroblasts co-cultured with 2500 μg mL^−1^ of supramolecular hydrogel γ-CDHSA3, where a high cell proliferation was observed. The cell viability study inferred that the supramolecular hydrogels synthesized in this work were biocompatible with low cell cytotoxicity. Therefore, γ-CDHSAs could be considered as safe drug delivery platform and is very promising for controlled drug delivery systems.

## 4. Conclusions

Through *in-silico* rational design it was possible to model different cross-linked PVA hydrogels containing γ-CD as platforms for sustained release of PDN. Using semi-empirical quantum mechanical calculations, the effectiveness of 20 carboxylic acids to generate a specific cross-linked hydrogel capable of supporting different amounts of γ-CD was evaluated. According to the interaction energies calculated, SA was selected as the candidate to carry out the crosslinking of the PVA hydrogel. MSD studies allowed the evaluation of the intermolecular interactions between PDN and three cross-linked hydrogel formulations with different proportions of γ-CD. As the γ-CD was added, the affinity of the drug for the hydrogel increased proportionally. γ-CDHSA3, which had the most γ-CDs in its structure, managed to capture between 26 and 27 PDN molecules. This would indicate that drugs interacted with both γ-CD and the PVA skeleton of the hydrogel.

A novel type of formulation used as a hydrophobic drug release platform was successfully synthesized and characterized. This formulation has been designed conjugating PVA, SA and incorporating γ-CD (γ-CDHSA). A series of three γ-CDHSAs varying the γ-CD content were described in order to elucidate its potential as a drug incorporation and delivery platform.

The γ-CDHSA hydrogels showed high PDN loading because of backbone affinity for the drug and the formation of possible inclusion complexes between PDN and γ-CD. The thermomechanical properties and ESR from the hydrogels can be regulated changing the γ-CD content. On the other hand, higher γ-CD content in the hydrogels was associated with a higher concentration of loaded PDN. At the same time, when the γ-CD content in the hydrogel increased, the release speed decreased. This could be due to multiple types of interactions involved in the complex formation of γ-CDHSAs with PDN, improving its stability. Also, it is important to note that increasing the γ-CD content increased the hydrophilicity of the hydrogels.

The loading process and release of PDN seems to be controlled by several factors, including the crosslinking degree, number and size of pores, γ-CD content, time of contact and types of intermolecular interactions that can be formed with the hydrogel. Moreover, the hydrogels showed good biocompatibility with L929 mouse connective tissue fibroblasts. In this study, the data concluded that stimuli-responsive hydrogels (swelling index) could change their volume significantly in response to small changes of certain environmental parameters such as time and pH. At physiological pH, it was observed that γ-CD content influenced the swelling index. In addition, the statistical analysis showed that γ-CD content influenced the percentage of PDN retention.

Taking into account that these formulations possess excellent mechanical properties, low cytotoxicity and they can be tunable according to the drug release requirements, these formulations may be utilized as an effective platform for drug incorporation and release to treat dermatological disease or other associated events.

## Figures and Tables

**Table 1:** Structure and properties of Prednisone (PDN) [9].

Chemical Structure	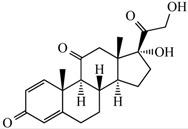
Molecular formula	C_21_H_26_O_5_
Appearance	Colorless crystals
Solubility in water (20 °C–25 °C)	Very slightly soluble
Mol. Wt.	358.434 g mol^−1^
Melting point	230–235 °C
Wavelength (λ, nm)	254 nm

**Table 2:** Characteristics of hydrogel formulations.

Hydrogel Formulation	γ-CD Proportion (%)	Copolymer Concentration % PVA/SA *w*/*w*	Hydrogel at 25 °C
γ-CDHSA1	2.44	20%	Yes
γ-CDHSA2	4.76	20%	Yes
γ-CDHSA3	9.1	20%	Yes

**Table 3:** Average interaction energy values calculated using semi-empirical quantum mechanics methods.

Id.	Hydrogel	Average Δ*E* kcal mol^−1^	Id.	Hydrogel	Average Δ*E* kcal mol^−1^
1	PVAc-Oxalic ac/γ-CD	−1.71 ± 0.05	11	PVAc-Adipic acid/γ-CD	−2.14 ± 0.03
2	PVAc-Malonic acid/γ-CD	−1.73 ± 0.07	12	PVAc-Pimelic acid/γ-CD	−2.20 ± 0.07
3	PVAc-Succinic acid/γ-CD	−2.67 ± 0.03	13	PVAc-Suberic acid/γ-CD	−2.23 ± 0.04
4	PVAc-Malic acid/γ-CD	−2.34 ± 0.08	14	PVAc-Azelaic acid/γ-CD	−2.40 ± 0.02
5	PVAc-Fumaric acid/γ-CD	−2.08 ± 0.04	15	PVAc-Phthalic acid/γ-CD	−2.54 ± 0.09
6	PVAc-Maleic acid/γ-CD	−1.98 ± 0.07	16	PVAc-Isophthalic acid/γ-CD	−2.60 ± 0.06
7	PVAc-Citraconic acid/γ-CD	−1.97 ± 0.04	17	PVAc-Terephthalic acid/γ-CD	−2.70 ± 0.05
8	PVAc-Itaconic acid/γ-CD	−1.88 ± 0.09	18	PVAc-2,5-pyridine acid/γ-CD	−2.40 ± 0.09
9	PVAc-Tartaric acid/γ-CD	−2.10 ± 0.03	19	PVAc-Aspartic acid/γ-CD	−2.83 ± 0.08
10	PVAc-Glutaric acid/γ-CD	−2.21 ± 0.05	20	PVAc-Glutamic acid/γ-CD	−2.63 ± 0.07

**Table 4:** Amount of PDN loaded into the γ-CDHSAs composite hydrogels. Results indicate the average (*n* = 3) ± standard deviation values. The same letters beside the standard deviation denotes the absence of statistical differences using Tukey HSD, at 95% confidence level).

Composite	Amount of Loaded PDN (mg g Dried Hydrogel^−1^) Concentration of Aqueous Soaking Solution 0.22 mg mL^−1^
γ-CDHSA1	8.36 ± 0.92
γ-CDHSA2	9.02 ± 1.23
γ-CDHSA3	10.1 ± 1.41

**Table 5:** PDN release percentage (The values between parenthesis are the codified values).

γ-CD Proportion (%)	Time of Release (h)	PDN Release Percentage (%)
0 (−1)	1 (−1)	14.01
0 (−1)	1 (−1)	13.90
0 (−1)	48 (1)	18.06
0 (−1)	48 (1)	28.02
2.44 (−0.4637)	8 (−0.70213)	65.77
2.44 (−0.4637)	8 (−0.70213)	63.42
2.44 (−0.4637)	8 (−0.70213)	68.69
2.44 (−0.4637)	8 (−0.70213)	69.43
2.44 (−0.4637)	8 (−0.70213)	62.62
2.44 (−0.4637)	8 (−0.70213)	63.33
4.76 (0.0462)	24.5 (0)	73.79
4.76 (0.0462)	24.5 (0)	75.53
4.76 (0.0462)	24.5 (0)	78.42
4.76 (0.0462)	24.5 (0)	80.09
4.76 (0.0462)	24.5 (0)	75.63
4.76 (0.0462)	24.5 (0)	78.20
9.10 (1)	1 (−1)	89.27
9.10 (1)	1 (−1)	85.60
9.10 (1)	48 (1)	98.89
9.10 (1)	48 (1)	99.41

## Supplementary Materials

The following are available online at http://www.mdpi.com/1999-4923/10/1/30/s1, Table S1: List of dicarboxylic acids evaluated as crosslinking agents in different PVA hydrogels; Table S2: Swelling experiments to determinate the influence of CD composition, pH and time.

Supplementary File 1
