# Supplementary material for: Rational Design, Synthesis and Evaluation of γ-CD-Containing Cross-Linked Polyvinyl Alcohol Hydrogel as a Prednisone Delivery Platform
Source: Pharmaceutics. 2018 Mar 7;10(1):30. doi: 10.3390/pharmaceutics10010030 (PMC5874843; doi:10.3390/pharmaceutics10010030)
Supplement: Supplementary File 1 [file pharmaceutics-10-00030-s001.pdf]

Supplementary Materials: Rational Design, Synthesis and Evaluation of γ-CD-Containing Cross-Linked Polyvinyl Alcohol Hydrogel as a Prednisone Delivery Platform

Adolfo Marican, Fabián Avila-Salas, Oscar Valdés, Sergio Wehinger, Jorge Villaseñor, Natalia Fuentealba, Mauricio Arenas-Salinas, Yerko Argandoña, Verónica Carrasco-Sánchez and Esteban F. Durán-Lara

Table S1. List of dicarboxylic acids evaluated as crosslinking agents in different PVA hydrogels

Id.	Crosslinking agent	Structure	References
1	Oxalic acid	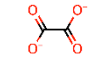	23
2	Malonic acid	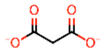	23-25
3	Succinic acid	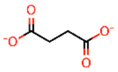	23,24,26,27
4	DL-Malic acid	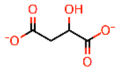	28
5	Fumaric acid	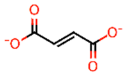	23,24
6	Maleic acid	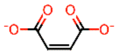	23,24
7	Citraconic acid	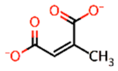	24
8	Itaconic acid	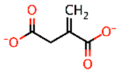	24
9	L-(+)-Tartaric acid	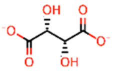	23
10	Glutaric acid	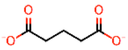	24,27,29,30
11	Adipic acid	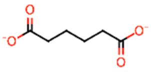	23,24,27,29
12	Pimelic acid	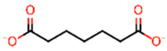	30
13	Suberic acid	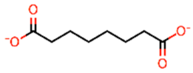	30
14	Azelaic acid	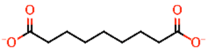	31
15	Phthalic acid	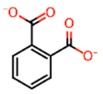	24, 32
16	Isophthalic acid	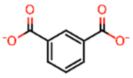	24
17	Terephthalic acid	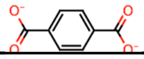	23,24,32

18	2,5-pyridine dicarboxylic	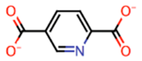	23
19	L-Aspartic acid	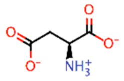	33
20	L-Glutamic acid	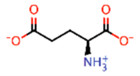	26, 33

Table S2. Swelling experiments to determine the influence of CD composition, pH and time. Numbers in parentheses indicate coded values.

Experiment	CD Composition [%]	pH	Time [h]	Swelling [%]
1	2.44 (-1)	4 (-1)	0 (-1)	0.0
2	2.44 (-1)	4 (-1)	1 (-0.957447)	132.0
3	2.44 (-1)	4 (-1)	2 (-0.914894)	115.6
4	2.44 (-1)	4 (-1)	3 (-0.872340)	219.7
5	2.44 (-1)	4 (-1)	4 (-0.829787)	189.3
6	2.44 (-1)	4 (-1)	5 (-0.787234)	215.6
7	2.44 (-1)	4 (-1)	6 (-0.744681)	198.4
8	2.44 (-1)	4 (-1)	7 (-0.702128)	184.4
9	2.44 (-1)	4 (-1)	24 (0.021277)	233.6
10	2.44 (-1)	4 (-1)	47 (1)	167.2
11	2.44 (-1)	7 (0)	0 (-1)	0.0
12	2.44 (-1)	7 (0)	0.1 (-0.996454)	66.7
13	2.44 (-1)	7 (0)	0.2 (-0.992908)	87.5
14	2.44 (-1)	7 (0)	0.3 (-0.985816)	89.0
15	2.44 (-1)	7 (0)	0.7 (-0.971631)	90.3
16	2.44 (-1)	7 (0)	1.2 (-0.950355)	91.2
17	2.44 (-1)	7 (0)	1.7 (-0.929078)	91.9
18	2.44 (-1)	7 (0)	2.2 (-0.907801)	91.5
19	2.44 (-1)	7 (0)	2.7 (-0.886525)	90.8
20	2.44 (-1)	7 (0)	3.2 (-0.865248)	91.7
21	2.44 (-1)	7 (0)	0 (-1)	0.0
22	2.44 (-1)	10 (1)	1 (-0.957447)	129.4
23	2.44 (-1)	10 (1)	2 (-0.914894)	154.2
24	2.44 (-1)	10 (1)	3 (-0.872340)	162.1
25	2.44 (-1)	10 (1)	4 (-0.829787)	141.8
26	2.44 (-1)	10 (1)	5 (-0.787234)	137.3
27	2.44 (-1)	10 (1)	6 (-0.744681)	135.0
28	2.44 (-1)	10 (1)	7 (-0.702128)	142.9
29	2.44 (-1)	10 (1)	24 (0.021277)	148.6
30	2.44 (-1)	10 (1)	47 (1)	184.7
31	4.76 (-0.298)	4 (-1)	0 (-1)	0.0
32	4.76 (-0.298)	4 (-1)	1 (-0.957447)	73.0
33	4.76 (-0.298)	4 (-1)	2 (-0.914894)	74.3
34	4.76 (-0.298)	4 (-1)	3 (-0.872340)	88.5
35	4.76 (-0.298)	4 (-1)	4 (-0.829787)	102.0
36	4.76 (-0.298)	4 (-1)	5 (-0.787234)	112.2
37	4.76 (-0.298)	4 (-1)	6 (-0.744681)	175.0
38	4.76 (-0.298)	4 (-1)	7 (-0.702128)	88.5
39	4.76 (-0.298)	4 (-1)	24 (0.021277)	107.4
40	4.76 (-0.298)	4 (-1)	47 (1)	86.5
41	4.76 (-0.298)	7 (0)	0 (-1)	0.0
42	4.76 (-0.298)	7 (0)	0.1 (-0.996454)	57.3
43	4.76 (-0.298)	7 (0)	0.2 (-0.992908)	75.6

44	4.76 (-0.298)	7 (0)	0.3 (-0.985816)	81.4
45	4.76 (-0.298)	7 (0)	0.7 (-0.971631)	81.8
46	4.76 (-0.298)	7 (0)	1.2 (-0.950355)	81.2
47	4.76 (-0.298)	7 (0)	1.7 (-0.929078)	82.4
48	4.76 (-0.298)	7 (0)	2.2 (-0.907801)	82.0
49	4.76 (-0.298)	7 (0)	2.7 (-0.886525)	82.0
50	4.76 (-0.298)	7 (0)	3.2 (-0.865248)	81.8
51	4.76 (-0.298)	10 (1)	0 (-1)	0.0
52	4.76 (-0.298)	10 (1)	1 (-0.957447)	140.9
53	4.76 (-0.298)	10 (1)	2 (-0.914894)	142.3
54	4.76 (-0.298)	10 (1)	3 (-0.872340)	206.0
55	4.76 (-0.298)	10 (1)	4 (-0.829787)	217.4
56	4.76 (-0.298)	10 (1)	5 (-0.787234)	225.5
57	4.76 (-0.298)	10 (1)	6 (-0.744681)	151.7
58	4.76 (-0.298)	10 (1)	7 (-0.702128)	281.9
59	4.76 (-0.298)	10 (1)	24 (0.021277)	228.9
60	4.76 (-0.298)	10 (1)	47 (1)	224.2
61	9.1 (1)	4 (-1)	0 (-1)	0.0
62	9.1 (1)	4 (-1)	1 (-0.957447)	131.8
63	9.1 (1)	4 (-1)	2 (-0.914894)	193.2
64	9.1 (1)	4 (-1)	3 (-0.872340)	153.4
65	9.1 (1)	4 (-1)	4 (-0.829787)	164.9
66	9.1 (1)	4 (-1)	5 (-0.787234)	170.3
67	9.1 (1)	4 (-1)	6 (-0.744681)	175.0
68	9.1 (1)	4 (-1)	7 (-0.702128)	123.0
69	9.1 (1)	4 (-1)	24 (0.021277)	145.3
70	9.1 (1)	4 (-1)	47 (1)	128.4
71	9.1 (1)	7 (0)	0 (-1)	0.0
72	9.1 (1)	7 (0)	0.1 (-0.996454)	64.9
73	9.1 (1)	7 (0)	0.2 (-0.992908)	74.4
74	9.1 (1)	7 (0)	0.3 (-0.985816)	73.4
75	9.1 (1)	7 (0)	0.7 (-0.971631)	76.1
76	9.1 (1)	7 (0)	1.2 (-0.950355)	76.4
77	9.1 (1)	7 (0)	1.7 (-0.929078)	76.1
78	9.1 (1)	7 (0)	2.2 (-0.907801)	74.6
79	9.1 (1)	7 (0)	2.7 (-0.886525)	75.9
80	9.1 (1)	7 (0)	3.2 (-0.865248)	75.7
81	9.1 (1)	10 (1)	0 (-1)	0.0
82	9.1 (1)	10 (1)	1 (-0.957447)	256.8
83	9.1 (1)	10 (1)	2 (-0.914894)	301.4
84	9.1 (1)	10 (1)	3 (-0.872340)	175.5
85	9.1 (1)	10 (1)	4 (-0.829787)	182.0
86	9.1 (1)	10 (1)	5 (-0.787234)	178.4
87	9.1 (1)	10 (1)	6 (-0.744681)	228.1
88	9.1 (1)	10 (1)	7 (-0.702128)	219.4
89	9.1 (1)	10 (1)	24 (0.021277)	223.0
90	9.1 (1)	10 (1)	47 (1)	275.5
